# Amniotic amputation

**DOI:** 10.11604/pamj.2015.21.90.6968

**Published:** 2015-06-05

**Authors:** Imene Dahmane Ayadi, Emira Ben Hamida

**Affiliations:** 1 Department of Neonatology, Charles Nicolle Hospital, Tunis El Manar University, Tunis, Tunisia

**Keywords:** Birth defect, amniotic band syndrom, constriction band

## Image in medicine

Amniotic band syndrome (ABS) is an uncommon, congenital fetal abnormality. Lower extremity limb defects are the common manifestations of ABS. The most common features include congenital distal ring constrictions, intrauterine amputations, and acrosyndactyly. Rare cases of craniofacial and visceral defects were reported. A female newborn, born at 33 weeks of gestation from a 43 years-old mother, gravida 6 para 6. The newborn was eutrophic (birth weight was 2500g, length was 46 cm and head circumference was 31 cm). Pregnancy was uneventful. Prenatal ultrasonography follow-up showed no abnormalities. Postnatal examination showed signs consistent with the diagnosis of amniotic bands syndrome at the newborn's left limbs. The newborn presented syndactyly, lymphedema, with distal agenesis of the 2^nd^, 3^rd^, and 4^th^ fingers; ring constrictions of the great toe, and distal agenesis of the 2nd toe. No other birth defect was associated. The newborn was discharged on the tenth day of life. He was referred for orthopedic management of these anomalies.

**Figure 1:**
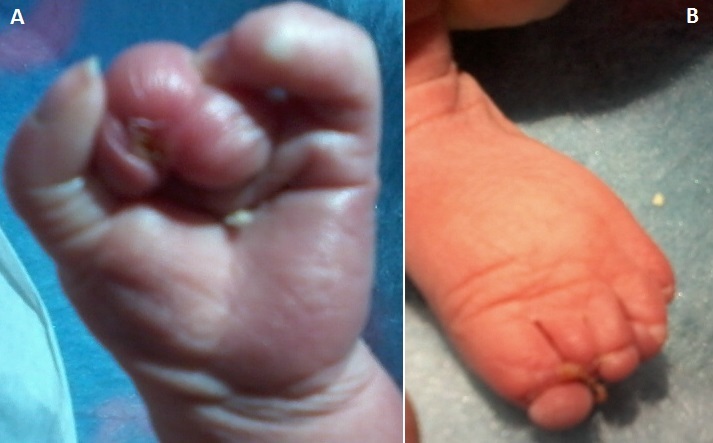
(A)amnotic amputation of fingers; (B) amniotic amputation and ring constrictions of toes

